# *Vibrio vulnificus* infection caused by a hand puncture wound from seafood: a case report

**DOI:** 10.3389/fmed.2026.1747587

**Published:** 2026-01-26

**Authors:** Lin Zhao, Peiting Lin, Tianyi Liu, Weidong Liu, Peilin Xie

**Affiliations:** 1 Department of Plastic Surgery, Gansu Provincial Hospital, Lanzhou, Gansu, China; 2 The First Clinical Medical College, Gansu University of Chinese Medicine, Lanzhou, Gansu, China

**Keywords:** antibiotic treatment, early diagnosis, surgical intervention, *V. vulnificus* infection, *Vibrio vulnificus*

## Abstract

*Vibrio vulnificus* infection is characterized by rapid progression and poor prognosis, which can lead to disability or death without timely intervention. In some cities or countries where the incidence of *V. vulnificus* infection is not high, it is easy to cause misdiagnosis and delay the treatment of the disease. This case showed a 42-year-old man from a non-coastal area in China who accidentally stabbed his right hand while handling seafood 1 day earlier. He developed significant local redness, swelling, pain, and systemic symptoms. When the patient visits the doctor, the doctor makes an accurate initial diagnosis based on the patient’s medical history and clinical manifestations. Effective antibiotic combination treatment is given before the feedback of bacterial culture results. Fasciotomy is immediately performed when there are signs of progression of osteofascial compartment syndrome. Finally, the patient’s right hand and right forearm were preserved.

## Introduction

*Vibrio vulnificus* is a bacterium naturally found in warm seawater, commonly present in coastal areas. *V. vulnificus* infection was first reported in 1979 (1). Since then, documented cases have confirmed its severity (2–4). Although infections are rare, they progress rapidly with high mortality rates, requiring prompt diagnosis and treatment to save lives (5). Here, we present a case of necrotizing fasciitis of the hand and cellulitis of the right forearm caused by *V. vulnificus* infection following a puncture wound from seafood. Through early administration of sensitive antibiotics and timely surgical intervention, we successfully cured the patient, providing crucial clinical reference for diagnosis and treatment.

## Case report

**FIGURE 1:**
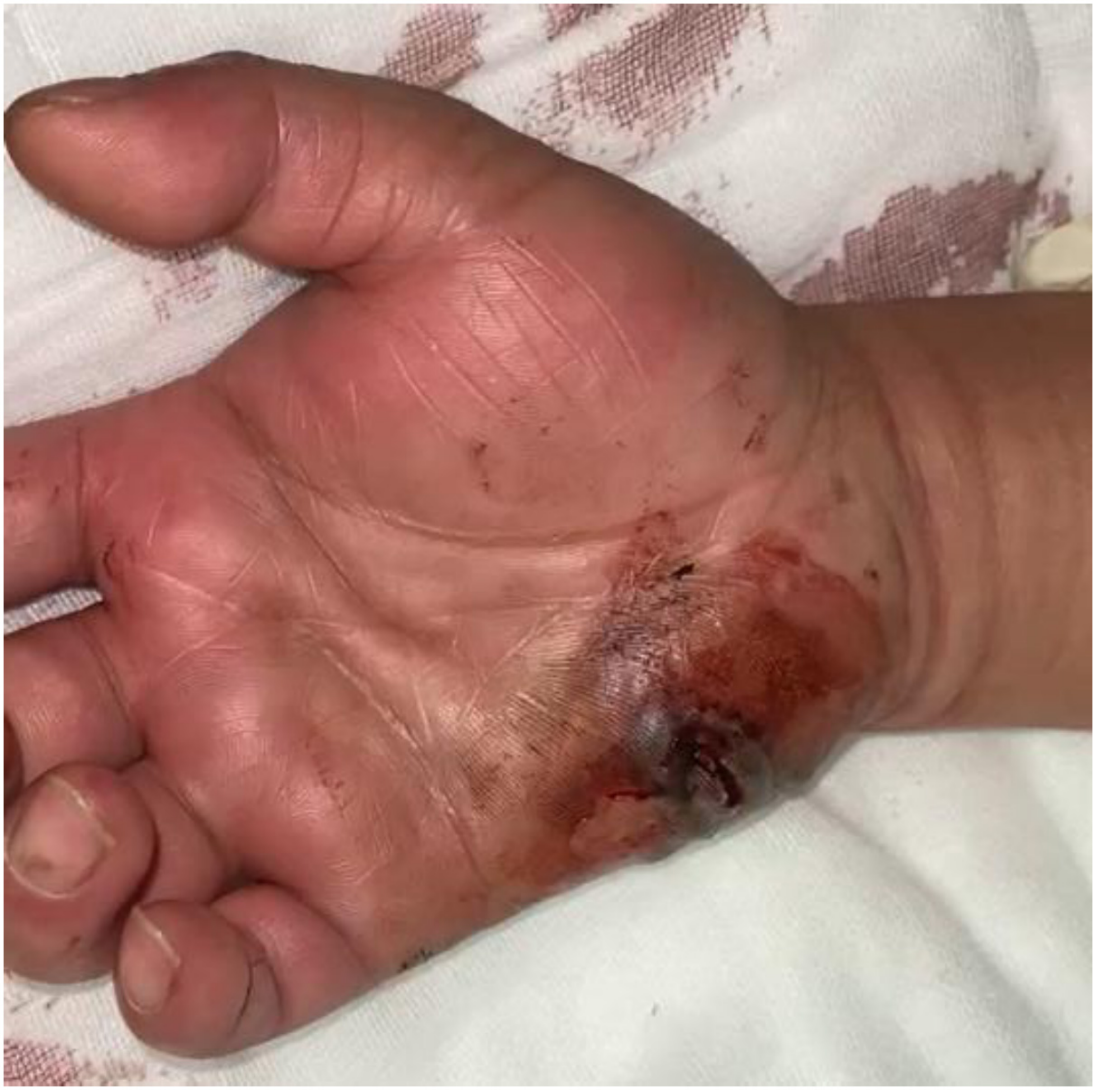
Clinical appearance of a full-thickness necrotic skin lesion in the hypothenar area of the right hand on admission.

A 42-year-old male was admitted to our department with a 1-day history of a lacerated wound and infection in his right hand. The patient reported sustaining a puncture injury to the hypothenar region of his right hand while handling seafood one day prior. The injury was initially associated with pain and bleeding, for which he received wound care at a local clinic. Subsequently, the wound developed progressive redness, swelling, and severe pain, rapidly spreading to the right forearm. Systemic symptoms, including chills, high fever, nausea, vomiting, and fatigue, and mild drowsiness. The patient presented emergently to our unit. On admission, the patient exhibited marked redness, swelling, and elevated skin temperature in the right hand and forearm, accompanied by severe pain. A full-thickness necrotic wound measuring approximately 3 cm × 2 cm was observed in the hypothenar region of the right hand (Figure 1). Based on the patient’s medical history and clinical presentation, a preliminary diagnosis of *V. vulnificus*-induced wound infection was made. Given the exceptionally high mortality rate associated with this condition, empirical antimicrobial therapy targeting Vibrio species (levofloxacin 500 mg IV qd, ceftriaxone 2 g IV q12h, and doxycycline 100 mg po q12h) was initiated immediately while awaiting the results of wound culture and sensitivity testing.

Admission laboratory results revealed:

- Interleukin-6 (IL-6): > 1,525.00 pg/mL (reference range < 7 pg/mL)
- Procalcitonin (PCT): 54.055 ng/mL (reference range < 0.065 ng/mL)
- White blood cell (WBC) count: 13.6 × 10^9^/L (reference range 3.5–9.5 × 10^9^/L)
- Neutrophil percentage: 93.20% (reference range 50.0%–75.0%)
- Neutrophil count: 12.67 × 10^9^/L (reference range 1.20–7.00 × 10^9^/L)
- Platelet count: 89 × 10^9^/L (reference range 100–300 × 10^9^/L)
- Glycated hemoglobin (HbA1c): 9.6% (reference range 4%–6%)
- Mean blood glucose: 12.8 mmol/L (reference range 3.6–7 mmol/L)
- Serum albumin: 28.30 g/L (reference range 40–55 g/L)
- Blood culture Gram stain: Gram-negative bacilli (positive)

**FIGURE 2:**
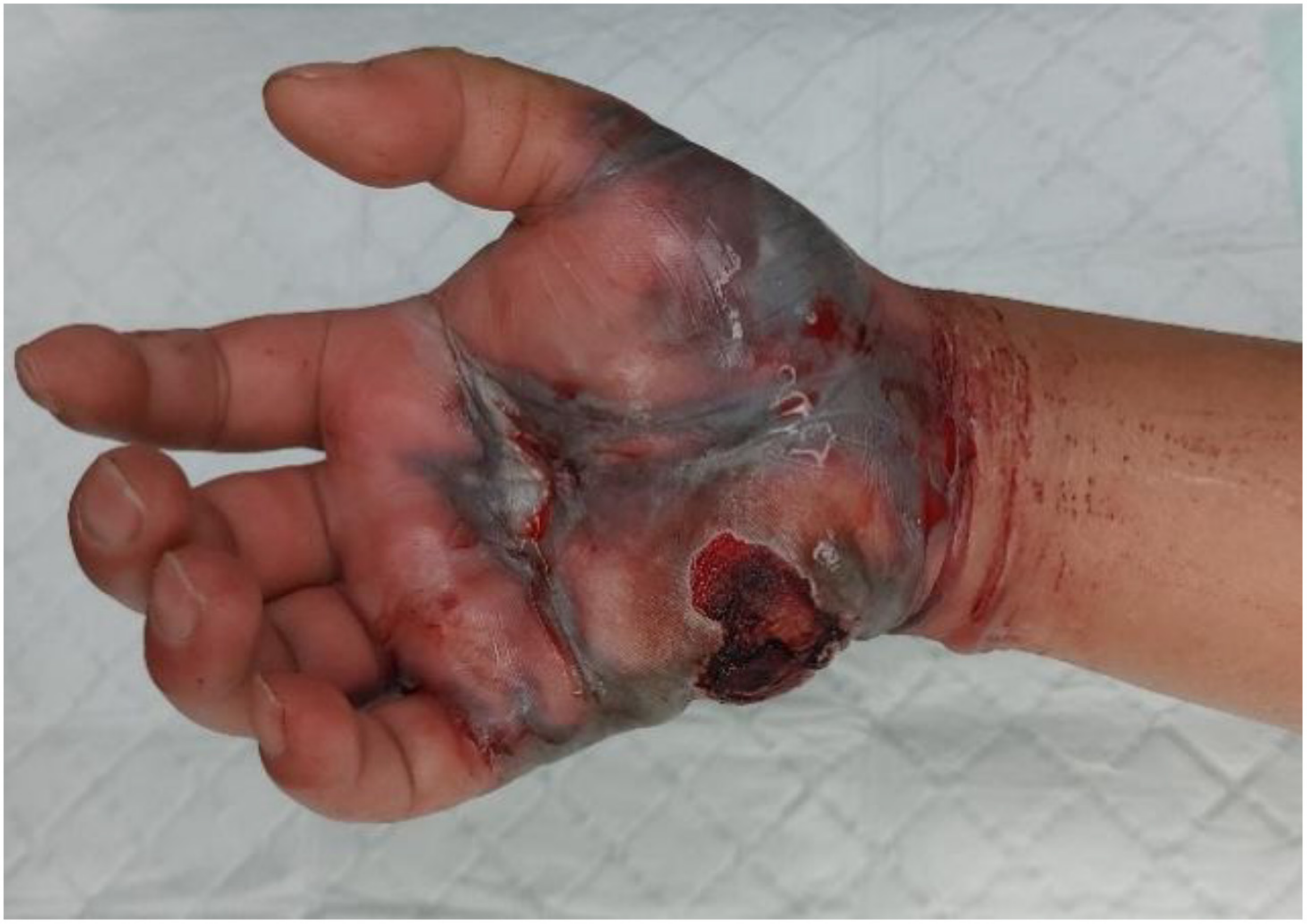
Clinical appearance of hemorrhagic bullae on the palmar side of the right hand.

**FIGURE 3:**
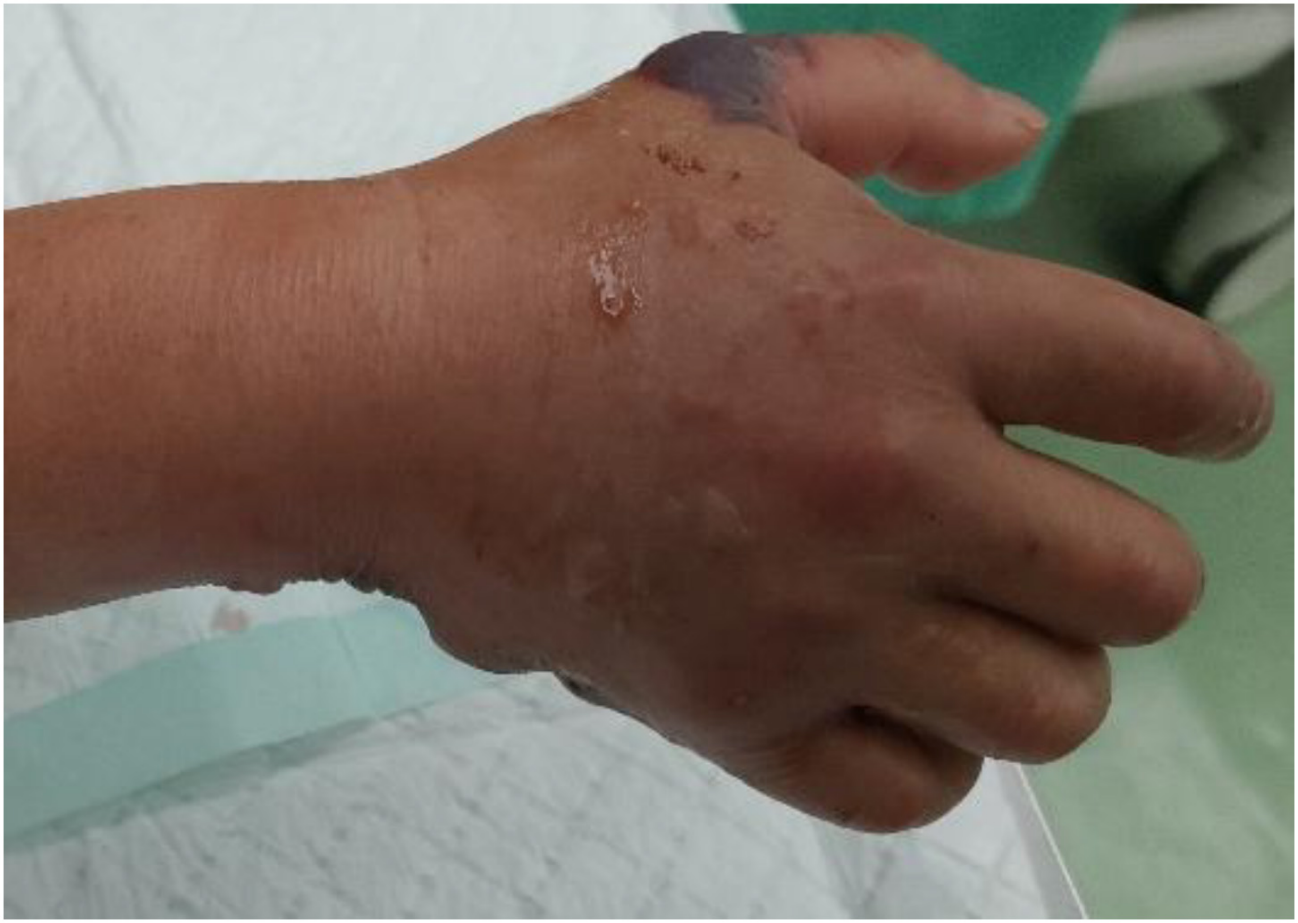
Clinical appearance of mottled and tense bullae on the dorsal side of the right hand.

**FIGURE 4:**
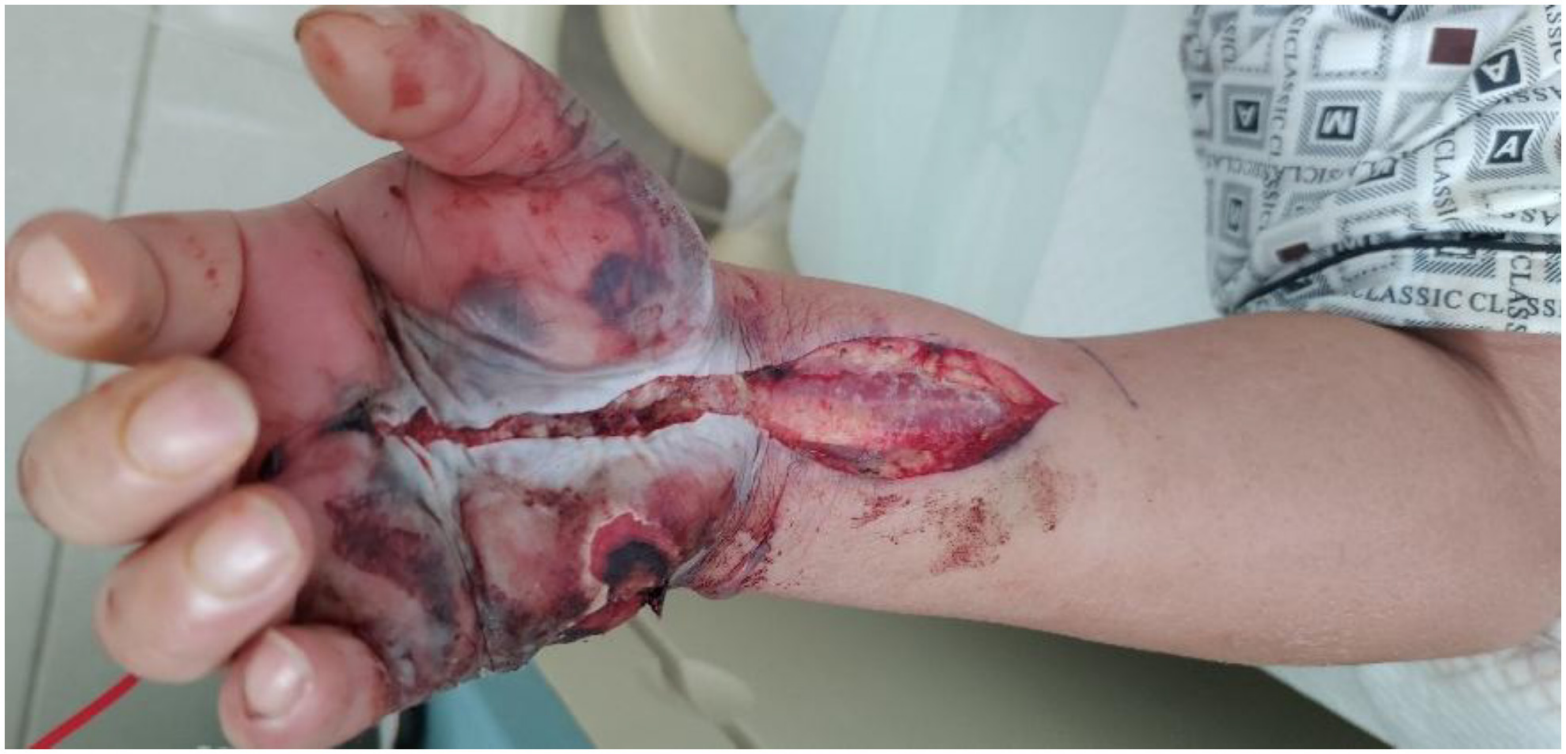
A palmar incision extending from the metacarpophalangeal (MCP) joint of the right middle finger to the distal one-third of the forearm.

**FIGURE 5:**
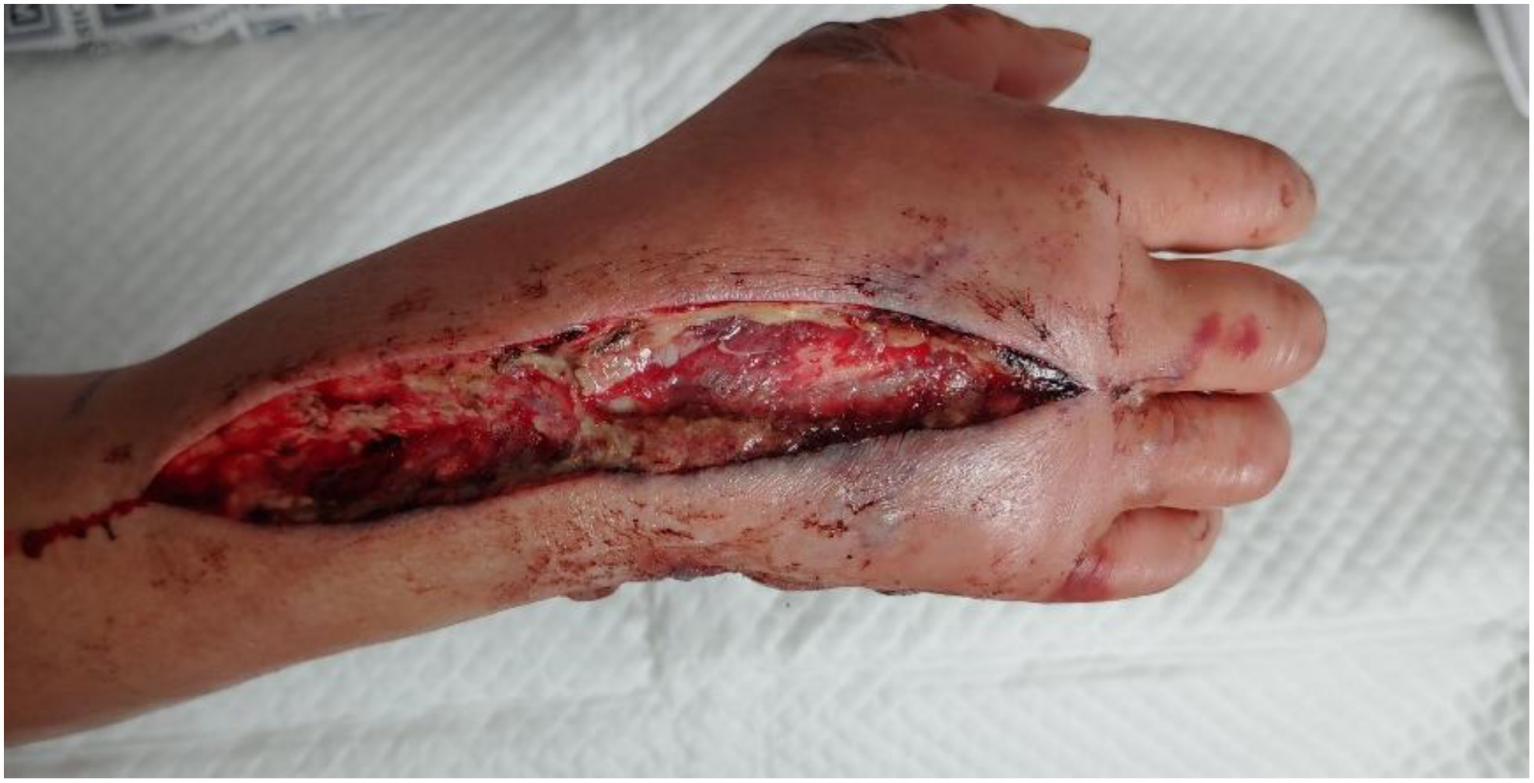
A dorsal incision extending from between the third and fourth metacarpophalangeal joints of the right hand to the distal one-third of the forearm.

By hospital day 2, the patient manifested a fulminant necrotizing soft tissue infection characterized by hemorrhagic bullae (Figure 2), ischemic mottling (Figure 3), and neurological deficits. When the patient exhibited worsening swelling in the right wrist and forearm, accompanied by numbness and a weak radial pulse, we informed the patient and his family about the severity of the condition. At this time, the patient had presented clinical manifestations of osteofascial compartment syndrome (pain, numbness, and weak radial pulse). In order to avoid serious consequences of disability caused by osteofascial compartment syndrome, the only effective treatment was emergency fasciotomy and decompression to release the high pressure and restore the blood supply to the limb. Emergency fasciotomy was performed with the following incisions (Figures 4, 5). During the dissection, strict attention was paid to preserving the integrity of blood vessels, nerves, and tendons. After achieving full decompression and thorough debridement, a negative pressure wound therapy system was applied. Postoperative Day 1 (Hospital Day 3): The patient remained in stable condition. And wound culture results confirmed *V. vulnificus* infection (Table 1). The current antibiotic regimen should be maintained, supplemented with targeted supportive care: limb elevation for edema control, intravenous fluid resuscitation, and high-protein nutritional support. On postoperative day 9, a secondary debridement was performed. The split-thickness skin grafting was performed on hospital day 21 and 44 to reconstruct the right hand defect. At last, the patient was discharged on hospital day 68. We recommend that the patient perform intensive right-hand functional exercises after discharge.

**TABLE 1:** Susceptibility profiles of *Vibrio vulnificus* isolates against tested antimicrobials.

Antibacterial drug	MIC	Result interpretation	Folding point standard		Antibacterial drug	MIC	Result interpretation	Folding point standard	
	μ g/mL		S	R		μ g/mL		S	R
Cefoperazone/sulbactam	1/8	–			Meropenem	≤ 0.125	S	≤ 1	≥ 4
Tetracycline	≤ 2	S	≤ 4	≥ 16	Cefoxitin	≤ 4	S	≤ 8	≥ 32
Chloramphenicol	≤ 4	S	≤ 8	≥ 32	Cefepime	8	SDD		
Trimethoprim/sulfamethoxazole	≤ 1/19	S	≤ 2	≥ 4	Ceftazidime	≤ 1	S	≤ 4	≥ 16
Levofloxacin	≤ 1	S			Cefuroxime	≥ 16	R	≤ 8	≥ 32
Ciprofloxacin	≤ 0.5	S			Cefazolin	8	R		
Amikacin	≤ 8	S	≤ 16	≥ 64	Piperacillin/tazobactam	≤ 4/4	S	≤ 16	≥ 128
Gentamicin	≤ 2	S	≤ 4	≥ 16	Ampicillin/sulbactam	≤ 4/2	S	≤ 8	≥ 32
Imipenem	≤ 0.25	S	≤ 1	≥ 4	Amoxicillin/clavulanic acid	≤ 8/4	S	≤ 8	≥ 32

In the table the third column shows the sensitivity of *V. vulnificus* to the antibacterial drug as sensitive (S), intermediate (I), and resistant (R). The sensitive antibiotics indicated in the table include tetracycline, levofloxacin and ceftazidime, which proved that doxycycline, levofloxacin and ceftriaxone used in our early stage were the right choices.

**FIGURE 6:**
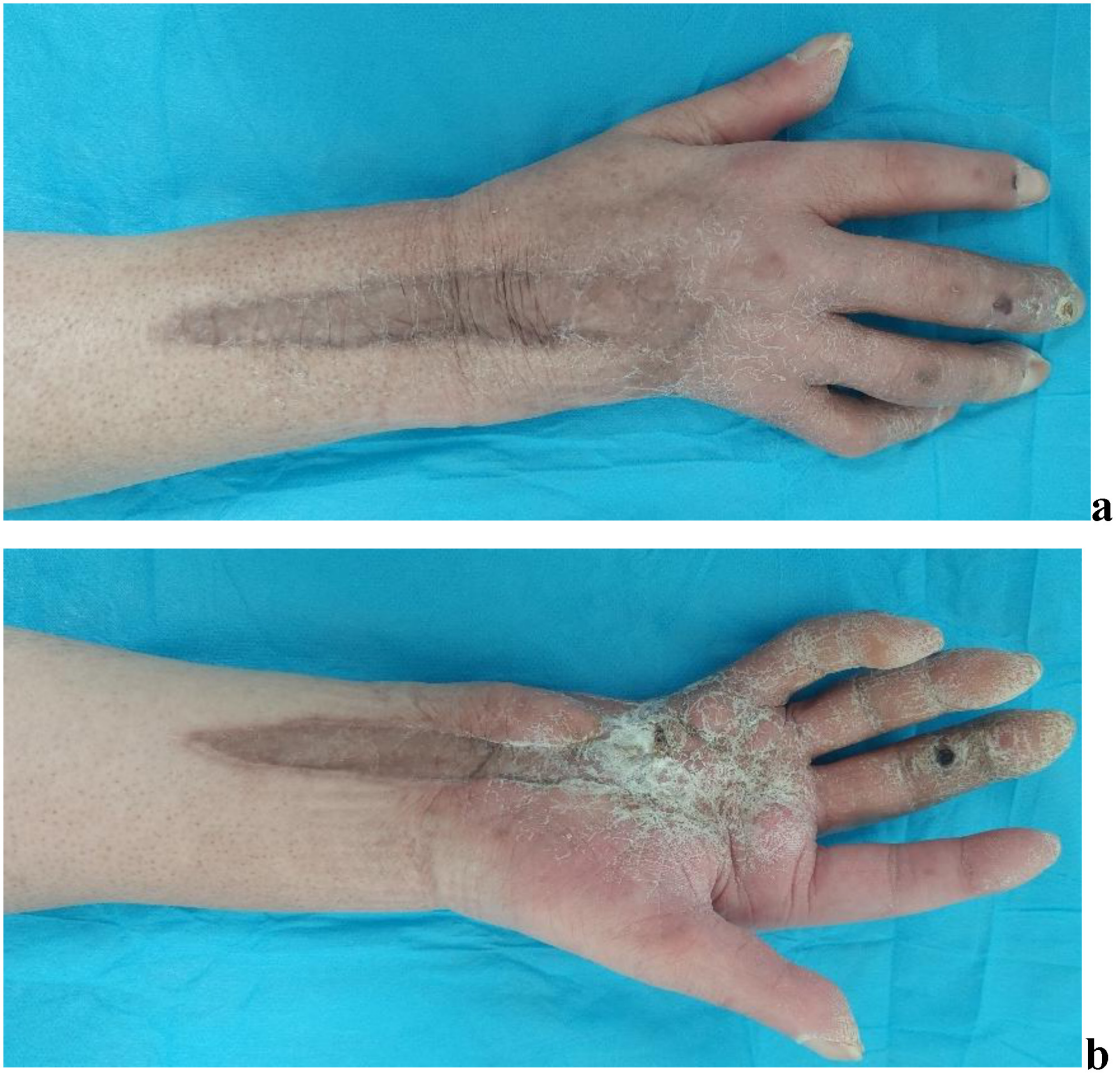
Performance of wound recovery of the patient 6 months after discharge. **(a)** Recovery of the wound on the dorsal side of the right hand and forearm. **(b)** Recovery of the wound on the palmar side of the right hand and forearm.

**FIGURE 7:**
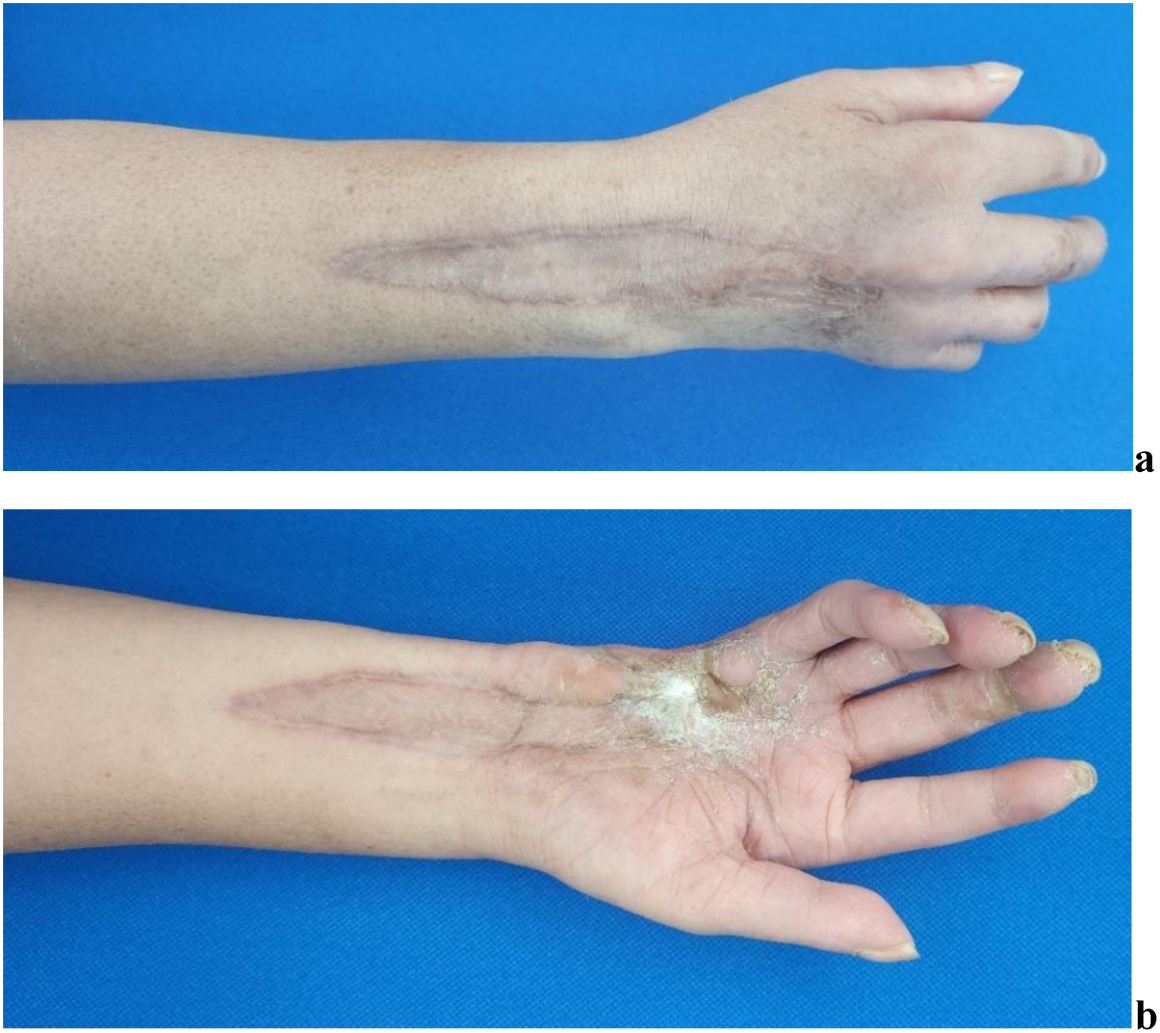
Performance of wound recovery of the patient 12 months after discharge. **(a)** Recovery of the wound on the dorsal side of the right hand and forearm. **(b)** Recovery of the wound on the palmar side of the right hand and forearm.

The patient attended scheduled outpatient follow-up visits in our department at 6 months (Figure 6) and 12 months (Figure 7) post-discharge. The scar tissue at the surgical site on the patient’s right hand and forearm has demonstrated progressive color and textural alignment with surrounding healthy tissue. The right wrist and digits 1–2 of the right hand demonstrate functional range of motion. But exhibiting moderately impaired flexion in the third digit, and significantly compromised both flexion and extension functions in the fourth and fifth digits.

## Discussion

*Vibrio vulnificus* is a bacterium found in brackish water. Human infection occurs primarily through the consumption of contaminated seafood or direct wound exposure to tainted water. *V. vulnificus* is a Gram-negative bacterium that was first isolated in 1964 by the US Centers for Disease Control and Prevention (6). It has the potential to cause severe, life-threatening infections in individuals who are at high risk or particularly susceptible.

*V. vulnificus* abundance and virulence are closely related to water temperature. The density of *V. vulnificus* increases slowly at low temperatures, rapidly at normal temperatures (18 °C to 26 °C), and stops increasing at high temperatures (7). Case reports of *V. vulnificus* infection in China are mainly concentrated in the southeastern coastal areas (such as Taiwan, Hainan, and Guangdong, etc.), where warm water conditions are most suitable for the survival of the pathogen (8). Among them, the average annual temperature in Taiwan is above 15 °C, but the infection rate of *V. vulnificus* is low between December and February, which is due to the decrease in fishing activities caused by the strong northeast monsoon that usually reaches Taiwan in winter (7). Hainan Province is located in the southernmost part of China. The province is characterized by high temperature and rain throughout the year, with no cold winters. According to a survey, among the foodborne bacteria contained in shellfish in Haikou seafood market throughout the year, the content of *V. vulnificus* was relatively high from July to November, and reached the highest value in September of the year (9). In regions of China with distinct four seasons, the detection of *V. vulnificus* in aquatic products is concentrated in summer and autumn (10). This is consistent with the study of Korean scholars, who found that *V. vulnificus* infections related to eating raw oysters were concentrated in August and September (11). Based on the above, it can be concluded that although there are large differences in temperature changes in four seasons in different regions of China, the infection of *V. vulnificus* is mainly concentrated in summer and autumn when the water temperature is warmer.

*V. vulnificus* infection is classified into three types: primary septicemia, wound infection and gastrointestinal diseases (6). Primary sepsis and wound infection can lead to serious complications. Clinically, they are often manifested as deep soft tissue infections, and most cases often develop into necrotizing fasciitis. In contrast, the gastrointestinal type is usually self-limiting. Primary sepsis is the most common clinical manifestation, occurring after the intake of raw seafood (such as raw oysters), and mainly affects individuals with potential comorbidities such as liver diseases (12, 13). Wound infection is a less common form of infection, such as when a patient is injured in a marine environment or a pre-existing wound is exposed to seawater (14).

The timeliness of treatment for *V. vulnificus* infection shows a significant positive correlation with case-fatality rates (15). Data indicate a 33% fatality rate with a 24-h delay in antibiotic therapy, escalating to 100% after 72 h (16, 17). Even with early and appropriate treatment, mortality rates remain persistently high (18). The standard antibiotic treatment protocol consists of doxycycline combined with a third-generation cephalosporin. Alternative therapeutic options include either a third-generation cephalosporin paired with a fluoroquinolone or fluoroquinolone monotherapy (19, 20). In a systematic review and meta-analysis published in 2024, the authors provided a broader antibiogram by synthesizing data from 32 articles across 13 Asian countries, covering 13 major antimicrobial groups against *V. vulnificus*. The results confirmed that tetracyclines, quinolones, and third-generation cephalosporins have lower antimicrobial resistance, highlighting their potential as primary treatment options (21). Antimicrobial therapy should adhere to the principles of early initiation, combination regimen, and adequate dosing (22). According to reports, the treatment of severe wound infections involves combination therapy with doxycycline/fluoroquinolones plus third-generation cephalosporins, along with aggressive surgical intervention (23).

Di et al. (24) reported a case of *V. vulnificus* infection caused by eating seafood 3 days ago. The patient developed typical skin lesions on both lower limbs within 12 h after admission, and rapidly progressed to primary sepsis. Despite antibiotic and surgical treatment, the patient finally died 16 days after admission. In this case, the patient was diagnosed as *Vibrio vulnificus* bloodstream infection caused by diet. The early symptoms did not attract the attention of the patient, and it was often late when the patient visited the hospital. Feng et al. (25) reported a case of a fisherman who was cut on the skin of his left upper limb while working on a fishing boat, and then came to the hospital for treatment because the skin was ulcerated for 1 week and did not heal. The patient had a large area of skin ulceration on his left upper limb when he was treated, and he was given combined treatment of antibiotics and surgery after admission, and finally he was cured and discharged. Fortunately, in this case, the patient’s condition did not progress very rapidly, so although he was admitted to the hospital one week after the injury, the wound was extensively eroded and eventually was successfully rescued, but this was only a case, and the vast majority of patients had lost the chance of treatment at this time. Zhang et al. (26) reported a case of a 69-year-old woman who visited the hospital 2 days after being stabbed by the dorsal fin of a live fish. The doctor gave antibiotics and surgical intervention, and finally the right little finger was removed due to dry gangrene.

Our case demonstrates a *V. vulnificus* infection secondary to a marine-product puncture wound. Upon presentation, the patient exhibited localized skin necrosis at the right-hand wound site, accompanied by systemic manifestations including chills, high fever, nausea, vomiting, fatigue and mild drowsiness. The infection rapidly progressed to extensive tissue destruction involving the right hand and forearm. Prior to receiving the bacterial culture results from the wound secretion, we accurately diagnosed the patient with *V. vulnificus* infection based on their history of seafood exposure and clinical manifestations. Immediately upon admission, combination therapy with levofloxacin, ceftriaxone, and doxycycline was initiated. When the patient exhibited worsening swelling in the right wrist and forearm, accompanied by numbness and a weak radial pulse, immediate decompressive incision was performed. Postoperative wound culture results confirmed *V. vulnificus* infection, and the antimicrobial susceptibility testing demonstrated that the bacterium was sensitive to all three selected antibiotics.

### Limitations of this case presentation:

During this treatment, we successfully saved the patient’s life and preserved their right hand, but due to fear of pain, the patient failed to adhere to postoperative rehabilitation exercises after skin grafting, resulting in partial loss of right-hand function.

## Conclusion

Throughout our patient’s treatment course, accurate preliminary diagnosis, early administration of sensitive antibiotics, and timely surgical intervention successfully saved the patient’s life while preserving the function of his right hand.

## Recommendations

*V. vulnificus* is an opportunistic human pathogen with a high mortality rate. To avoid *V. vulnificus* infection, people can prevent it by avoiding raw or undercooked seafood in their diet. Patients with open wounds (such as recent surgery, skin piercings, or tattoos) should avoid contact with the marine environment (do not swim or fish, etc.) and should not handle raw seafood with their bare hands. The above situation should be highly vigilant in any season, especially in the summer and autumn when the incidence is high. In clinical diagnosis and treatment, doctors need to combine the patient’s medical history and clinical manifestations, early treatment with sensitive antibiotics and timely surgical intervention for suspected *V. vulnificus* infection patients, which are the key to reduce disability and mortality.

## Data Availability

The original contributions presented in this study are included in this article/supplementary material, further inquiries can be directed to the corresponding author.
